# The Duty on Medical Supplies

**Published:** 1888-03

**Authors:** 


### The Duty on Medical Supplies.

At the annual meeting of the Georgia Medical Society, held on January 3, 1888, the following resolution was unanimously adopted:

*Resolved*, That the Corresponding Secretary enter into correspondence with other State and local Medical Societies, with the view to induce them to act in concert with us in an effort to influence Congress to remove the *Import Duty* from all medical and surgical supplies, instruments, and appliances.

Certainly all will agree that "a feeling of humanity suggests that all medical and surgical supplies, instruments, and appliances, including those used in the diagnosis as well as in the treatment of diseases, should be furnished to those needing them at the lowest possible price." Could our Government be persuaded to remove this duty, the public as well as the physicians would be the gainers. It is a fact that a large proportion of the medical instruments and appliances offered to the medical profession is of poor quality, and it is just as much a fact that good instruments cost too much. For these reasons many physicians are prevented from supplying themselves with even a moiety of the instruments required for general practice. It is a well-known fact that the majority of English and German made medical instruments and supplies are *better* than those made in America, and it is just as well known that such supplies can be bought at a much lower price abroad than in this country. We need not stop to inquire into the causes of this further than to say that the chief cause is the import duty on such supplies, by which the makers of such supplies in this country have the physicians and the public at their mercy. Nor do we purpose writing on "free trade" or "protection," further than to say that the best protection a country can give its citizens is to take measures to protect their health.

If foreign made medical supplies were placed on the free list, the quality and prices of those foreign articles would compel our home makers to lower their prices and improve the quality of their products—there would then be no market for poor instruments.

The Georgia Medical Association has acted wisely in bringing this matter directly to the individual members of the profession. The only people that can possibly be hurt by the carrying out of the suggestion are the manufacturers; but even if they are injured, it is demanded for the greatest good of the greatest number.

The above will apply equally to *Dental,* as well as Medical Instruments and supplies. Does not the cause of suffering humanity, require that those things employed especially for the mitigation of its ills, have free course and admission, duty free to all countries of the world, wherever there is demand for them?

ED. REG.

